# *Ab Initio* Approach to Second-order Resonant Raman Scattering Including Exciton-Phonon Interaction

**DOI:** 10.1038/s41598-017-07682-y

**Published:** 2017-08-04

**Authors:** Yannick Gillet, Stefan Kontur, Matteo Giantomassi, Claudia Draxl, Xavier Gonze

**Affiliations:** 1 Institute of Condensed Matter and Nanosciences, Nanoscopic Physics, European Theoretical Spectroscopy Facility, Université catholique de Louvain, Chemin des étoiles 8, bte L7.03.01, 1348 Louvain-la-Neuve, Belgium; 2 Physics Department and IRIS Adlershof, Humboldt-Universität zu Berlin, and European Theoretical Spectroscopy Facility, Zum Großen Windkanal 6, D-12489 Berlin, Germany

## Abstract

Raman spectra obtained by the inelastic scattering of light by crystalline solids contain contributions from first-order vibrational processes (e.g. the emission or absorption of one phonon, a quantum of vibration) as well as higher-order processes with at least two phonons being involved. At second order, coupling with the entire phonon spectrum induces a response that may strongly depend on the excitation energy, and reflects complex processes more difficult to interpret. In particular, excitons (i.e. bound electron-hole pairs) may enhance the absorption and emission of light, and couple strongly with phonons in resonance conditions. We design and implement a first-principles methodology to compute second-order Raman scattering, incorporating dielectric responses and phonon eigenstates obtained from density-functional theory and many-body theory. We demonstrate our approach for the case of silicon, relating frequency-dependent relative Raman intensities, that are in excellent agreement with experiment, to different vibrations and regions of the Brillouin zone. We show that exciton-phonon coupling, computed from first principles, indeed strongly affects the spectrum in resonance conditions. The ability to analyze second-order Raman spectra thus provides direct insight into this interaction.

## Introduction

In Raman spectroscopy, the change of frequency and intensity of light, that is inelastically scattered by the material, allows one to collect a rich set of information, even more if the effect is monitored as a function of the frequency of the incoming light^1–4^. In most systems, the energy loss is due to the creation of one or more phonons, and different orders of phonon-photon scattering processes contribute to the scattering cross section. The first-order contribution, in which only one phonon is emitted or absorbed, yields information on vibrations with very low crystalline momentum, due to the negligible momentum of light. Extracting information on phonon frequencies from the first-order spectrum is straightforward, as clear selection rules govern the appearance or extinction of usually well-isolated peaks. In contrast, second-order Raman processes exhibit features coming from different crystalline momenta, potentially from the entire Brillouin zone (BZ), with the only constraint of negligible momentum of the phonon pairs involved in the two-phonon process. They are more complex and, hence, more difficult to interpret. When the frequency of the incoming light comes close to the specific frequency needed to drive the transfer of an electron from an occupied state to an unoccupied state, which is termed a resonance, the absolute Raman intensities can change by several orders of magnitude. This resonance phenomenon appears independently of the number of emitted phonons. Thus a particular frequency can be selected to increase the Raman signal^3^. The possibility to create bound electron-hole pairs, called excitons, crucially impact optical absorption and related photocurrent in many materials, a mechanism at work in commercial photovoltaic devices^5–8^. Understanding and characterizing their dynamics and interactions is an experimental challenge. In this context, the ability to interpret the widely available Raman data to characterize exciton-phonon interaction is crucial for the understanding of light-matter interaction in general, and in particular in technologically relevant materials.

Experimentally, the second-order part of the Raman spectrum is often used to characterize low-dimensional or layered materials, such as e.g. graphene^9^ or graphite^10^, and more recently also transition-metal dichalcogenides^11–15^. The few existing first-principles studies for these systems relied on the independent-electron approximation (IPA)^16, 17^, in which the formation of excitons is neglected. As these low-dimensional materials exhibit very well-defined and specific spectral features^18^, their second-order Raman spectra can still be interpreted quite easily, at least concerning the location of the peaks. However, for photovoltaic applications, bulk materials form the vast majority of relevant materials. Their Raman spectra contains a larger number of features, and is thus difficult to decipher. Resonant first-order and second-order Raman scattering processes may have e.g. different temperature behavior^19^, whose most natural interpretation invoke characteristics of the direct and indirect gaps. For classical semiconductors such as silicon (and Ge, III-V etc), the analysis of second-order Raman spectra was facilitated by the use of simple Hamiltonians, based on the effective mass approximation or on semi-empirical pseudopotentials, with model phonon band structure, model electron-phonon interaction and, in some cases, the inclusion of Wannier-Mott excitons^20–24^. Early first-principles calculations were done for vanishing laser frequencies (no resonance effects) without excitonic effects^25^. Our work allows one to compute from first-principles second-order Raman spectra without any of these limitations.

**Figure 1:**
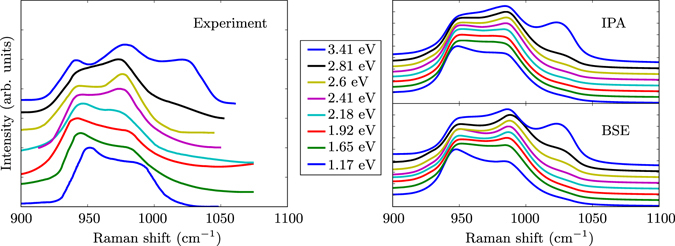
Experimental (left, taken from ref. 26) and theoretical (right) second-order Raman spectra of silicon, between 900 and 1050 cm^−1^, for different excitation energies between 1.17 eV and 3.41 eV, normalized to their maximal values. For clarity, the curves are shifted with respect to each other (lowest excitation energy at the bottom). Theoretical results have been obtained in the independent-particle approximation (IPA) and in the Bethe-Salpeter equation (BSE) formalism.

A prototypical example of resonant second-order Raman spectra is presented in Fig. 1. It shows the experimental frequency-dependent Raman spectra of silicon between 900 and 1050 cm^−1^, as measured by Renucci and coworkers^26^. The spectra are characterized by significant variations in intensity depending on the frequency of the incoming light, even for frequencies corresponding to energies smaller than the direct gap of 3.2 eV. Such resonance effects are allowed in second-order Raman processes, due to indirect transitions between electronic states that are caused by phonons of finite wavevector. Hence, it is clear that multi-phonon processes beyond first order are crucial. Below, we will show that all the features can be well interpreted by our first-principle theoretical approach.

A general methodology for describing multi-phonon scattering processes up to arbitrary order has been proposed by Knoll and Draxl^27^, but its application had been long restricted to the *ab initio* calculations of first-order resonant Raman scattering, in the IPA, without excitonic effects, e.g. in various superconducting materials^28–30^ and ladder compounds^31–33^. Recently, thanks to the treatment of excitons using the Bethe-Salpeter equation (BSE), excitonic effects were included from first principles in such first-order resonant Raman scattering^34^. Excitonic effects were clearly observed in the absolute intensity enhancement, and proved crucial to obtain good agreement with experimental data. Other first-principles studies of Raman intensities have been based on density-functional perturbation theory^35–42^. However, this last approach has been developed only in the limit of vanishing light frequency, so without addressing any resonance effect and without excitonic effects^25^.

In this work, we address the even more challenging computation of second-order resonant Raman scattering, including exciton-phonon coupling. Even by today standards, the computational load is quite formidable. We compute the ingredients using state-of-the-art methodology (density-functional perturbation theory and Bether-Salpeter equation), while also showing the adequacy of the simplifying “overtone” approximation. Applying it to the case of Si, we not only reproduce the above described experimental data, but give insight into the origin of the spectral features, and explore the role of excitonic effects on the Raman intensities.

## Results

We first describe the *normalized* intensities for Raman shifts in the 900–1100 cm^−1^ range. Such results are presented in the right panel of Fig. 1. On both levels of theory, IPA and BSE, the evolution of the intensity of the Raman shift with increasing light frequency (or photon excitation energy) follows very closely the experimental data. While most of the spectral weight is localized around 950 cm^−1^ for the lowest excitation energy of 1.17 eV, the shoulder below 1000 cm^−1^ is getting more pronounced with increasing excitation energy and starts to dominate above 2.4 eV. A third peak around 1030 cm^−1^ that is hardly visible in the bottom curves, is particularly pronounced only for the highest excitation energy of 3.41 eV. In excellent agreement with experiment, its intensity is nearly the same as the peak at 950 cm^−1^, and somewhat lower than the one at 980 cm^−1^.

**Figure 2:**
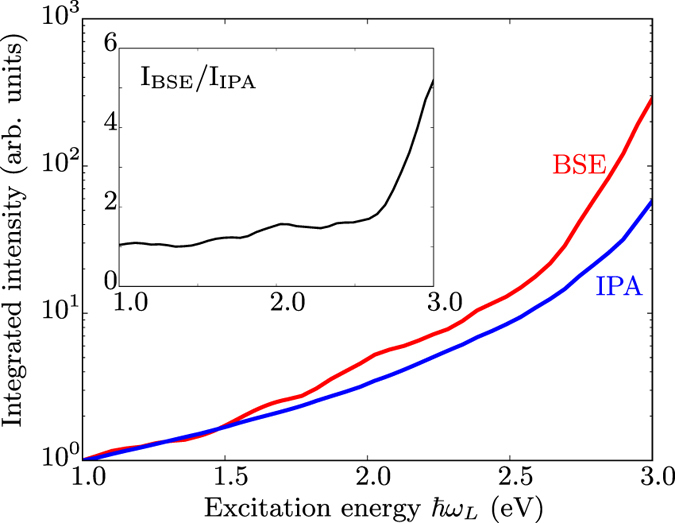
Integrated intensities of BSE and IPA spectra over Raman shifts ranging from 900 cm^−1^ to 1050 cm^−1^, as a function of the excitation energy $\hslash {\omega }_{L}$. The ${({\omega }_{L}-{\omega }_{R})}^{4}$ prefactor, see Eq. 1 has not been included, and the spectrum has been renormalized with respect to the value at $\hslash {\omega }_{L}=\mathrm{1.0\ }$eV. Note the logarithmic scale of the intensity. The inset shows the ratio between integrated intensities obtained by the IPA and the BSE.

Second, we examine the *integrated* intensities. Figure 2 shows a comparison between the IPA and the BSE absolute intensity, divided by ${({\omega }_{L}-{\omega }_{R})}^{4}$ (see Methods section for more detail), integrated over the range between 900 and 1100 cm^−1^, as a function of the excitation energy. Even if in this range of frequencies the relative intensities computed in the two formalisms are very similar as shown in Fig. 1, their absolute intensities differ by a factor 5 for excitation energies around 3 eV, as exhibited in the inset of the Fig. 2. Clearly, excitonic effects are important, which will be analyzed in the Discussion section.

**Figure 3:**
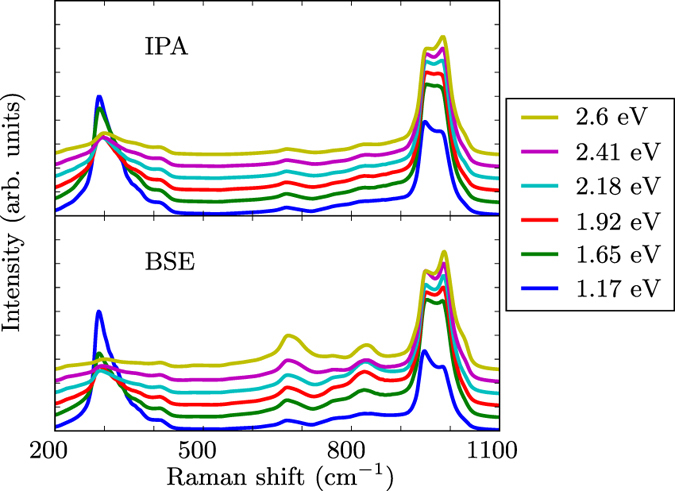
Second-order Raman spectra calculated within the IPA (top) and the BSE (bottom) for several excitation energies $\hslash {\omega }_{L}$. Each spectrum is normalized with respect to its highest value, as in Fig. 1.

The Raman spectrum in a broader range of Raman shifts is also obtained. In addition to the enhancement of the absolute intensity, similar to the one shown in Fig. 2, Fig. 3 shows that the use of the BSE, that is, the inclusion of exciton-phonon coupling, results in a pronounced intensity increase in the part of the Raman spectrum between 600 and 900 cm^−1^, especially for excitation energies between 2 and 3 eV. This additional excitonic effect will also be discussed later.

**Figure 4:**
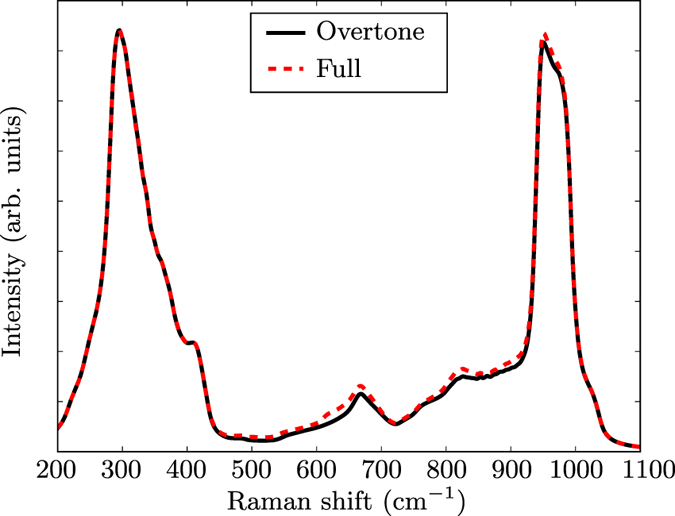
Second-order Raman spectrum obtained by differentiating the dielectric constant (at $\hslash {\omega }_{L}=0$) obtained within density-functional perturbation theory. “Full” means that the emission of all possible phonon pairs are considered in the calculation, while for the “overtone” graph, only the emission of time-reversed phonon pairs are taken into account.

In order to compute these results, we have made the approximation that the second-order contribution to Raman spectrum is dominated by the emission of two phonons that are connected by time-reversal symmetry: same frequency, and time-reversed collective eigendisplacement and momentum. From a group-theoretical point of view, derivatives with respect to such phonon pairs will always be active and contribute to the fully symmetric component of the spectrum^3^. However, group-theoretical arguments do not insure the vanishing of other contributions for arbitrary phonon wavevectors. While this approximation is justified a posteriori by the agreement show in Fig. 1, it has been nevertheless possible to theoretically examine its validity, in the specific case of vanishing excitation energy. We tested this approximation by computing the second-order derivatives of the dielectric constant obtained within Density-Functional Perturbation Theory (DFPT), where the electric field is used as a perturbation^43^. In this approach, the excitation energy vanishes, but unlike in the IPA, the local-field effects are taken into account, which makes its level of sophistication intermediate between IPA and BSE. Figure 4 shows the dominance of the derivatives by the overtone pair of modes (by around one order of magnitude with respect to other contributions in the worst case), which fully justifies our approach. This ‘overtone’ approximation was a crucial step in making the BSE computation tractable, as discussed in the Methods section, and to be able to treat excitonic effects. The confirmation of the validity of the ‘overtone’ approximation for silicon, discussed in prior works, also for other simple semiconductors^4, 20, 21, 23–26, 44–46^ is thus another significant result of our work. However, there are several cases in which features can be assigned to combination modes, like in refs 16, 47 and 48.In such case, the identification of relevant combinations might be done in the static limit using DFPT, followed by the computation of the contributions from specific mode combinations in the relevant energy spectrum (as is done already in our work, for degenerate phonon modes).

## Discussion

Our first-principles theory can provide insight into the origin of the features seen in Fig. 1, and then can shed light on the underlying physics, especially the role of excitonic effects. For this purpose, we first present the expression that yields the theoretical Raman intensity ***I*** in Fig. 1 (right part), 2, and 3. For a Raman shift $\hslash {\omega }_{R}$, at light excitation energy $\hslash {\omega }_{L}$, ***I*** is as follows:

1 $${I}^{\alpha \beta }({\omega }_{R},{\omega }_{L})=\frac{{\hslash }^{2}{({\omega }_{L}-{\omega }_{R})}^{4}}{{\mathrm{(4}\pi )}^{2}{c}^{4}}\frac{1}{{N}_{q}}\sum _{m{\bf{q}}}\{{[\frac{{\partial }^{2}{\varepsilon }^{\alpha \beta }({\omega }_{L})}{\partial {U}^{m{\bf{q}}}\partial {U}^{m(-{\bf{q}})}}\frac{n({\omega }_{m{\bf{q}}})+1}{2{\omega }_{m{\bf{q}}}}]}^{2}\delta ({\omega }_{R}-2{\omega }_{m{\bf{q}}})\}\mathrm{.}$$

The tensorial nature of ***I*** (*α* and *β* referring to the polarization of incoming and outgoing light) will not play an important role in our discussion. Apart prefactors, the Raman intensity ***I*** is essentially obtained by summing over contributions from all phonons, characterized by their momentum **q** and branch index *m*. Such contributions are products of the *change of the dielectric response* $\varepsilon$ due to each phonon and its time-reversed homolog, by a function that depends *only on the phonon frequency* ${\omega }_{mq}$ and a Dirac-delta, that expresses the conservation of energy. This formula is explained in more detail in the Methods section, which provides its derivation, including the “overtone” approximation, as well as the description of dielectric response calculations, with or without excitonic effects.

In Eq. (1), the Dirac-delta function, integrand of the two-phonon density of states

2 $${g}_{2DOS}({\omega }_{R})=\frac{1}{{N}_{q}}\sum _{m{\bf{q}}}\delta ({\omega }_{R}-2{\omega }_{m{\bf{q}}})$$

is actually weighted by a function of the phonon mode and excitation energy (the term in square brackets in Eq. (1))^2, 4^. One can expect such weighting factor to be a reasonably smooth function of the wavevector **q**. By contrast, the Dirac-delta function of Eq. 2 needs to be properly integrated throughout the Brillouin zone. It induces van Hove singularities, that dominate the overall shape of ***I***.

**Figure 5:**
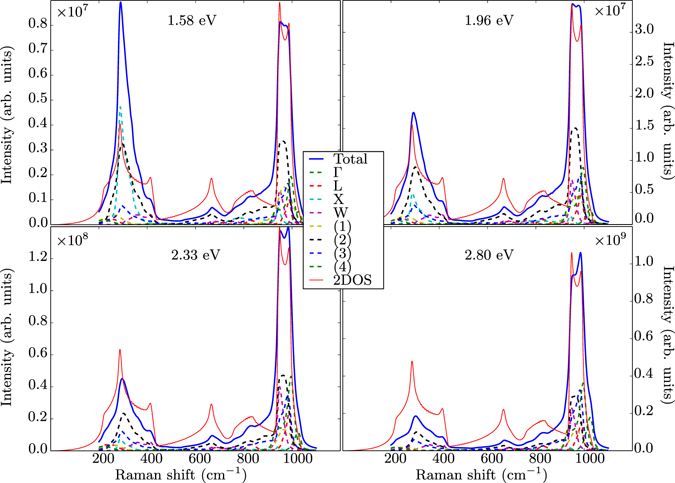
Diagonal component of the second-order Raman spectrum obtained in the IPA (blue lines), and its wavevector decomposition for four different excitation energies $\hslash {\omega }_{L}$: 1.58 eV, 1.96 eV, 2.33 eV and 2.80 eV. Eight **q** wavevectors are considered, in reduced coordinates: Γ = (0, 0, 0), L = (1/2, 1/2, 1/2), X = (1/2, 1/2, 0), W = (1/4, 1/2, 3/4), (1) = (1/4, 1/4, 0), (2) = (1/2, 1/4,0), (3) = (−1/4, 1/4, 0), (4) = 1/4, 1/4, 1/4). The two-phonon density of states is also represented for sake of comparison (red lines).

**Figure 6:**
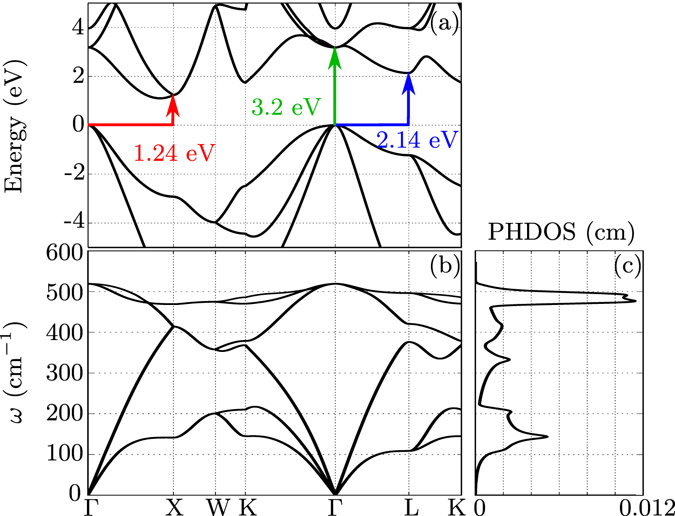
(**a**) Electronic Kohn-Sham band structure of silicon, with a scissor shift of 0.65 eV for the conduction bands. Possible direct transitions at Γ and indirect transitions from Γ to X and from Γ to L are indicated by the green, red, and blue arrows, respectively. (**b**) Phonon band structure and (**c**) phonon density of states.

**Figure 7:**
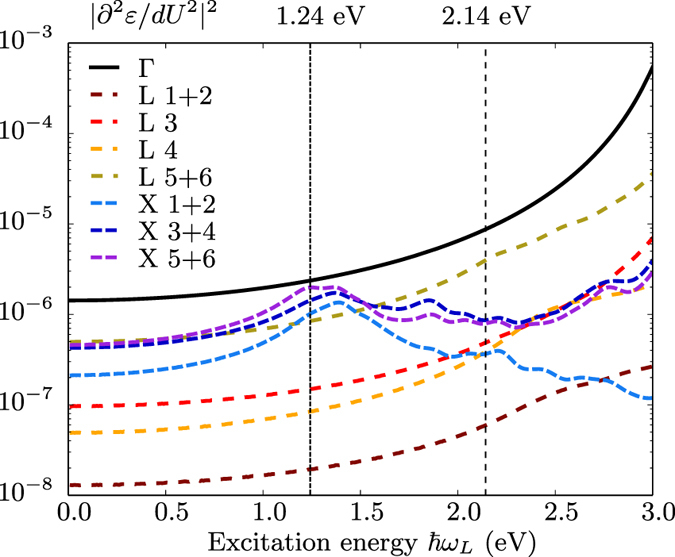
Second derivatives of the IPA dielectric function with respect to the excitation energy for different modes at the Γ, *X*, and *L* points. The notation “**q** m” represents the ${m}^{{\rm{th}}}$ branch of a q-point (Γ, *X*, or *L*), while “q *m* + *n*” represents the sum of the corresponding derivatives of the degenerate branches *m* and *n* at this point. The electronic transition thresholds for *X* (1.24 eV) and *L* (2.14 eV) are also indicated.

The second-order Raman scattering intensity can thus be decomposed into contributions coming from different points of the Brillouin zone for phonons. The assignment of peaks or characteristic features to selected phonons for Si had been established already in the seventies^44, 45^. Our methodology now allows one to examine accurately the behaviour of the intensity of each feature as a function of the light excitation energy $\hslash {\omega }_{L}$, especially close to the resonance, as first attempted in the nineties by Grein and Cardona^23^ using the theoretical and computational tools available at that time. This is shown in Fig. 5, where the evolution of the diagonal component of the IPA Raman spectrum with respect to the excitation energy is depicted for eight characteristic wavevectors in the Brillouin zone. In this framework, ‘optical’ transitions between different electronic wavevectors, i.e., between **k** and **k** + **q** with non-zero **q** are forbidden in the equilibrium structure. The phonon vibrations, however, may change some of these transitions from forbidden to allowed, giving rise to non-zero momentum matrix elements, thus leading to finite second-order derivatives of the dielectric function with respect to the phonon displacement. As a reference, the electronic and phononic band structures are shown in Fig. 6, along with the one-phonon density of states (related to the two-phonon density of state given in Eq. (2), by a simple scaling of the phonon frequency axis). As shown in Fig. 6(b), at the *X* point, the highest phonon frequency is about 470 cm^−1^, twice this value being 940 cm^−1^; the electronic resonance energy is 1.24 eV. At the *L* point, twice the highest phonon frequency is slightly larger around 1000 cm^−1^, with a resonance at 2.14 eV. Likewise, for the Γ point, the corresponding values are 1040 cm^−1^ and 3.2 eV. From a careful inspection of Fig. 5, we can conclude that the peak structures in Fig. 1 are related to these resonances. To highlight their behavior as a function of the excitation energy, we plot in Fig. 7 the second derivatives of the dielectric function at various points of the BZ. At the *X* point, it shows a clear maximum at the energy of the indirect band gap. The four curves related to the *L* point do not peak at their resonance frequency, but increase with excitation energy, with a reduced rate above 2 eV. The curve at Γ grows unabated even beyond 3.0 eV. From such different characteristics, one can also conclude that the two-phonon density of states alone is not sufficient to describe second-order Raman spectra in general, and even more so in resonance condition.

**Figure 8:**
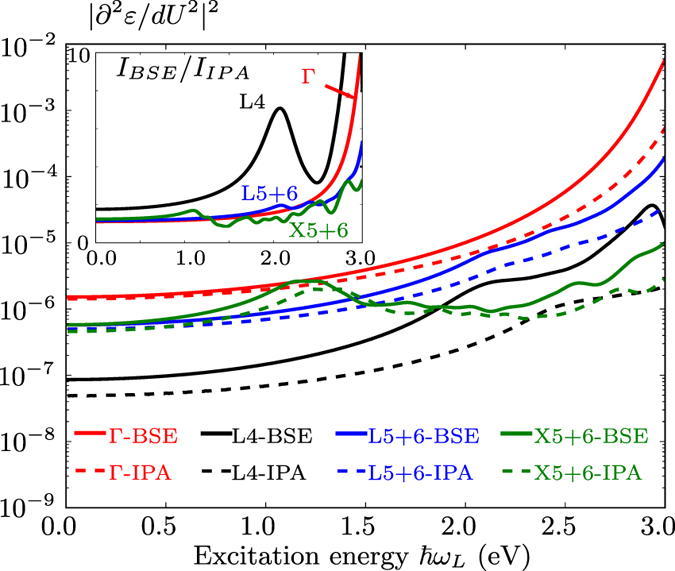
Second derivatives of the dielectric function with respect to the excitation energy for the highest-energy modes at Γ, *X* and *L* as well as the fourth mode at the *L* point. The inset shows the ratio between the BSE and the IPA results.

To assess the impact of excitonic effects, we have performed the corresponding calculations with the derivatives of the dielectric function for phonon wavevectors Γ, *X*, and *L* obtained by solving the BSE. A comparison with the IPA results is given in Fig. 8, where we emphasize the logarithmic scale of the vertical axis. The corresponding Raman spectra obtained for several excitation energies are presented in Fig. 3. As a general trend, the ratio between the BSE and IPA intensities (highlighted in the inset) increases with the excitation energy, exhibiting a maximum at the direct band gap. This effect is similar to the first-order enhancement effect reported in ref. 34. A very strong excitonic effect can be observed between 2 and 3 eV for the third and fourth mode of the *L* point, as depicted in Fig. 8 for the latter. It can be explained by the presence of bound exciton states (inside the gap) with finite momentum^49^ that become visible at second order due to the presence of finite-momentum phonons. The ratio between BSE and IPA results dramatically increases in this energy range due to the changed onset of the second-order response. The other *L*-point modes, around 100 and 500 cm^−1^, do not exhibit strong coupling with excitons. Overall, exciton-phonon coupling results in a pronounced intensity increase in the part of the Raman spectrum which is dominated by *L* modes, for excitation energies between 2 and 3 eV and Raman shifts between 600 and 900 cm^−1^ (Fig. 3). This result is a central outcome of the present work.

For the other modes, indirect transitions appear less important for the resonance behavior than direct transitions. For example, in the regions around 1000 and 1200 cm^−1^, all the intensities increase with energy with a similar rate. Since the relative intensities are well reproduced within the IPA, this explains why the IPA spectra in this range are in already quite good agreement with experiment (see Fig. 1 for a comparison between IPA and BSE in this range). On the other hand, an accurate description of absolute intensities requires the inclusion of excitonic effects as they lead to changes of one order of magnitude for excitation energies of around 3 eV, as already shown in Fig. 2.

Silicon, a paradigmatic material in solid-state physics, might however not be the material in which exciton-phonon coupling has the biggest impact on the second-order Raman spectrum. Indeed, for materials that possess infra-red active phonon modes (unlike silicon), the Fröhlich coupling with electrons diverges for small wavevectors, and dominates the exciton-phonon interaction. This has been explored for the second-order Raman case on the basis of model Hamiltonians in the eighties and nineties, see e.g. ref. 24 and references therein. The present approach can be generalized to the treatment of such Fröhlich coupling^50^.

## Conclusions

Thanks to a new method that combines frozen-phonon supercell calculations of dielectric functions and their derivatives with phonon spectra from density-functional perturbation theory, we have been able to compute frequency-dependent second-order Raman spectra. We have applied our approach to the computation of the second-order Raman intensities of silicon for a series of excitation energies. We not only reach excellent agreement with measured spectra^26^ but provide insight into the resonance behavior through analysis in terms of electronic structure and contributions from phonons at different points of the Brillouin zone. Excitonic effects turn out to be crucial to determine absolute Raman intensities as already found for the first-order scattering^34^. Moreover, we have shown that strong excitonic effects may also appear selectively for some vibrational modes of different phonon wavevectors, modifying the relative intensities of different peaks. Our approach leads to a deeper understanding of the physics involved in second-order Raman processes and facilitates the interpretation of experimental results. While in this article we have focused on silicon–a simple but prototypical material–our methodology is generally applicable to a broad range of materials.

## Methods

We focus first on the theoretical methods, and then we give the computational details.

In the harmonic approximation for the vibrational properties of periodic solids, the following expression can be used to compute the Raman spectrum^21, 27^ for two Stokes processes:

3 $$\begin{array}{c}{I}^{\alpha \beta }({\omega }_{R},{\omega }_{L})=\frac{{({\omega }_{L}-{\omega }_{R})}^{4}{\hslash }^{2}}{{\mathrm{(4}\pi )}^{2}{c}^{4}}\frac{1}{{N}_{q}}\sum _{{\bf{q}}}\sum _{{m}_{1},{m}_{2}}\{{[\frac{{\partial }^{2}{\varepsilon }^{\alpha \beta }({\omega }_{L})}{\partial {U}^{{m}_{1}{\bf{q}}}\partial {U}^{{m}_{2}(-{\bf{q}})}}]}^{2}\frac{1}{4{\omega }_{{m}_{1}{\bf{q}}}{\omega }_{{m}_{2}(-{\bf{q}})}}\\ \quad \quad \quad \quad \quad \,\,\,\times [{n}_{{m}_{1}{\bf{q}}}+\mathrm{1][}{n}_{{m}_{2}(-{\bf{q}})}+\mathrm{1]}{\delta }_{\gamma }({\omega }_{R}-({\omega }_{{m}_{1}{\bf{q}}}+{\omega }_{{m}_{2}(-{\bf{q}})}))\},\end{array}$$

where *α* and *β* are Cartesian indices related to the polarization of the incoming and outgoing light, **q** are phonon wavevectors sampling the full BZ homogeneously (with a total of *N*_*q*_ points), *m*_1_ and *m*_2_ are phonon branch indices, ${\omega }_{R}$ is the spectral frequency (Raman shift), ${\omega }_{L}$ is the excitation frequency, *c* the speed of light, ${\omega }_{m{\bf{q}}}$ is the phonon frequency of the ${m}^{{\rm{th}}}$ phonon branch at **q**, ${n}_{m{\bf{q}}}$ is the temperature-dependent phonon occupation factor ${n}_{m{\bf{q}}}={({e}^{\frac{\hslash {\omega }_{m{\bf{q}}}}{kT}}-1)}^{-1}$ and *δ*_*γ*_ is a Dirac-delta function replaced by a Lorentzian function of width *γ* for numerical purposes. In Eq. (3), the derivative of the dielectric function, *ε*, is obtained through an expansion of the dielectric function with respect to the phonon eigendisplacements. Extracting the second-order process from this expansion and using the adiabatic approximation^27^ gives, for the electronic part, the following second-order derivative:

4 $$\frac{{\partial }^{2}{\varepsilon }^{\alpha \beta }}{\partial {U}^{{m}_{1}{\bf{q}}}\partial {U}^{{m}_{2}(-{\bf{q}})}}=\sum _{\kappa \alpha l,\kappa ^{\prime} \beta l^{\prime} }\frac{{\partial }^{2}{\varepsilon }^{\alpha \beta }}{\partial {R}_{\kappa \alpha }^{l}\partial {R}_{\kappa ^{\prime} \beta }^{l^{\prime} }}{e}^{i{\bf{q}}({{\bf{R}}}_{l}-{{\bf{R}}}_{l^{\prime} })}{U}_{\kappa \alpha }^{{m}_{1}{\bf{q}}}{U}_{\kappa ^{\prime} \beta }^{{m}_{2}(-{\bf{q}})},$$

where **R**_*l*_ is the coordinate of the ${l}^{{\rm{th}}}$ periodic cell, ${R}_{\kappa \alpha }^{l}$ the coordinate of the ${\kappa }^{{\rm{th}}}$ atom along *α* axis in the ${l}^{{\rm{th}}}$ periodic cell. ${U}_{\kappa \alpha }^{m{\bf{q}}}$ are the atomic eigendisplacements of the dynamical problem, following the conventions of ref. 43.

The numerical evaluation of Eq. 3 is highly challenging since it requires careful integration over the Brillouin zone and summation over two mode indices. This contrasts with the summation over only one mode index, at the zone center, required for first-order Raman intensities.

We now describe how to numerically evaluate from first-principles the above-mentioned equations. A full *ab-initio* phonon band structure can be obtained from DFPT^38, 41^. As a first step, the phonon frequencies ${\omega }_{m{\bf{q}}}$ and the phonon eigendisplacements ${U}_{\kappa \alpha }^{m{\bf{q}}}$ are computed for a set of homogeneous **q**-points, and interpolated on a denser mesh of wavevectors thanks to the knowledge of interatomic force constants, as described in ref. 43. Unfortunately, existing DFPT implementations do not provide second derivatives of the frequency-dependent dielectric function with respect to atomic positions especially when excitonic effects are to be included. We thus numerically differentiate the dielectric function with respect to the eigendisplacements, which requires supercells of different size, depending on the phonon wavelength. In the frozen-phonon approach, a supercell, compatible with the selected wavevector **q** is generated: the axes of this supercell $\{{\bf{R}}{\text{'}}_{j}\}$ fulfill the constraint ${e}^{i{\bf{q}}{\bf{R}}{^{\prime} }_{j}}=1$, which means that **q** is a reciprocal vector of the supercell. The atoms of the supercell are then displaced with real displacements defined from the phonon eigendisplacements and phase factors

5 $${u}_{\kappa \alpha l}\triangleq \frac{1}{2}[{U}_{\kappa \alpha }(-{\bf{q}}){{\boldsymbol{e}}}^{-{\boldsymbol{i}}{\bf{q}}{{\bf{R}}}_{l}}+{U}_{\kappa \alpha }(-{\bf{q}}){{\boldsymbol{e}}}^{-{\boldsymbol{i}}{\bf{q}}{{\bf{R}}}_{l}}],\quad {v}_{\kappa \alpha l}\triangleq \frac{-i}{2}[{U}_{\kappa \alpha }({\bf{q}}){e}^{{\boldsymbol{i}}{\bf{q}}{{\bf{R}}}_{l}}-{U}_{\kappa \alpha }(-{\bf{q}}){{\boldsymbol{e}}}^{-{\boldsymbol{i}}{\bf{q}}{{\bf{R}}}_{l}}]\mathrm{.}$$

In the supercell, **k** + **q** points of the Brillouin zone are folded back to **k** points of the supercell. This allows for probing indirect transitions.

The calculations of the optical spectrum can be performed with different levels of accuracy, ranging from the independent particle approximation to time-dependent DFT (TDDFT) and, finally, many-body perturbation theory (MBPT) with the inclusion of excitonic effects by means of the Bethe-Salpeter equation^51, 52^.

We now detail why even for a small system like silicon such calculations are rather demanding (extremely demanding in the BSE case). First, for any wavevector, the summations over the ${N}_{modes}$ phonon modes in Eq. 3 require ${N}_{modes}\times {N}_{modes}$ evaluations of the derivatives. If the dielectric tensor is evaluated on a grid of 5 × 5 pairs of displacements, in order to get the second-order derivatives, the number of calculations required for a single wavevector in the BZ becomes 25 × 6^2^ = 900 evaluations of the dielectric tensor for silicon. In contrast, for first-order calculations, the number of computations for the zone-center wavevector amounts to 5 evaluations for each mode, thus 5 × 6 = 30 evaluations of the dielectric function to reach the final Raman spectrum of silicon. This number can be even reduced to only 5 by using symmetry arguments: acoustic modes do not contribute and the three other modes are degenerate.

It should also be noted that, unlike first-order spectra, second-order resonant Raman scattering requires the use of supercells commensurate with the wavevectors, and the computational cost increases quickly with the size of the supercell. Indeed, the number of bands included in the calculations scales linearly with the number of atoms. In the case of IPA, the number of electronic transitions scales linearly with the product of the number of valence and conduction bands and linearly with the number of k-points. Taking into account the folding of bands in the case of supercell, and the computational cost required for the dipole matrix elements, the overall scaling is roughly cubic with the size of the supercell. In BSE, however, the kernel introduces coupling between transitions, and the number of matrix elements in the transition basis set scales with the square of the product of the number of valence and conduction bands, and with the square of the number of k-points. Because of the reduction of the number of k-points with larger supercells, the number of matrix elements therefore increases with the square of the size of the supercell. Since the evaluation of the screened Coulomb matrix elements requires a double sum over planewaves, we conclude that the ratio between a BSE run for a supercell and the one for a unit cell increases as the fourth power of the number of replicas in the supercell. Finally, as already observed in ref. 34, the derivatives of the dielectric function are highly sensitive to the sampling of the BZ, leading to very demanding calculations in the case of BSE. Even if the frozen-phonon methodology requires a large amount of evaluations of dielectric functions, we have succeeded to apply the technique to three-dimensional materials by using approximations and numerical techniques as follows.

As mentioned in the Results section, the so-called “overtone” contributions, i.e., considering only derivatives coming from twice the same phonon mode, except when degenerate, should largely dominate other second-order contributions. In Eq. (3) this corresponds to neglecting all terms where ${\omega }_{{m}_{1}}\ne {\omega }_{{m}_{2}}$ and yields Eq. (1).

The overtone approximation allowed us to reduce significantly the number of required computations. Eventually, only one-dimensional derivatives are needed for non-degenerate modes, reducing the number of evaluations required for one wavevector from 900 for the full expression to 5 × 6 = 30. For high-symmetry points of the BZ, few two-dimensional derivatives are still required between two different modes belonging to the same degenerate subspace. As shown in Fig. 1(b), the frequency-dependent spectrum is well reproduced for silicon with this approximation. This figure corresponds to the $Z(XX)\overline{Z}$ scattering configuration.

In order to further reduce the computational load, the second-order derivatives of the dielectric function have been evaluated only on a coarse mesh of phonons wavevectors, while the phonon frequencies, the occupation numbers and Dirac functions have been calculated via DFPT techniques and summed on a dense mesh. This is equivalent to taking an averaged density of states around the coarse points as the density function of ref. 27.

The corresponding grid parameters are now described, as well as other computational details. We use the ABINIT package^53, 54^, with Troullier-Martin pseudopotentials (available from the ABINIT package^55^). Relaxed cell parameters of 10.20 Bohr are used with cut-off values for the wavefunctions of 8 Hartree. The DFPT phonons displacements and frequencies are interpolated from a coarse Γ-centered phonon grid of 8 × 8 × 8 to a non-shifted 70 × 70 × 70 mesh, to obtain the phonon density of states, following ref. 43, while a 4-times shifted 8 × 8 × 8 sampling is used for the electrons.

When simulated in the IPA (without local-fields effects)^56^, the dielectric function is evaluated for different supercells corresponding to a non-shifted 4 × 4 × 4 phonon wavevector grid (containing 64 wavevectors), while for the electronic states, the equivalent of a centered 24 × 24 × 25 grid for the primitive cell is used. In the supercell case, the Brillouin zone is folded, and the integration grid adapted accordingly. The convergence has been checked on the final Raman spectrum with respect to a 4-times shifted 2 × 2 × 2 centered phonon wavevector grid (containing 32 wavevectors). A scissors operator of 0.65 eV is applied on top of the Kohn-Sham eigenvalues to mimic the opening of the gap by GW.

In order to assess the importance of excitonic effects, we have performed BSE calculations of the derivatives of the dielectric function for phonons with Γ, *X* and *L* wavevectors (corresponding to a 2 × 2 × 2 Γ-centered grid). Dielectric functions obtained on 12 × 12 × 12 wavevectors in the BZ have been averaged following the technique described in ref. 34 to reach samplings equivalent to 24 × 24 × 24. 3 valence bands and 6 conduction bands were used for the primitive cells, their numbers being increased proportionally with the number of atoms in the supercells. The model dielectric function described in ref. 57 with ${\varepsilon }^{\infty }=12$ has been used to calculate the screening matrix elements. A cut-off energy of 4 Ha is used to describe the screening matrix in reciprocal space.

In order to present a Raman spectrum obtained with a sufficient number of phonon frequencies in the BSE framework, we have interpolated the ratio between BSE and IPA intensities towards a non-shifted 4 × 4 × 4 k-point grid with a trilinear interpolation in reduced coordinates and used these ratios to correct the IPA intensities.

When integrated intensities are presented, the ${({\omega }_{L}-{\omega }_{R})}^{4}$ factor is discarded. This factor is present also in the Raman spectrum of reference wide-band gap materials, like $Ca{F}_{2}$, to which the comparison is made in order to establish the absolute intensity. Hence, it can be discarded. Other factors are expected to be constant in wide band-gap materials.

## References

[1] Yu, P. Y. & Cardona, M. *Fundamentals of Semiconductors: Physics and Material Properties* (Springer, 2010).

[2] Weber, W. & Merlin, R. *Raman Scattering in Materials Science* (Springer, 2000).

[3] Cardona, M. & Guntherodt, G. *Light scattering in solids/edited by M. Cardona; with contributions by M.H. Brodsky… [et al.]* (Springer-Verlag Berlin; New York, 1975).

[4] Cardona, M. & Guntherodt, G. *Light scattering in solids II/edited by M. Cardona; with contributions by M. Cardona… [et al.]* (Springer-Verlag Berlin; New York, 1982).

[5] Lanzani, G. *The Photophysics behind Photovoltaics and Photonics* (Wiley, 2012).

[6] Pan, Z. *et al*. Polarization-resolved spectroscopy imaging of grain boundaries and optical excitations in crystalline organic thin films. *Nat. Commun*. **6**, doi:10.1038/ncomms9201 (2015). 10.1038/ncomms9201 PMC4579592 26365682

[7] Akselrod, G. M. *et al*. Visualization of exciton transport in ordered and disordered molecular solids. *Nat. Commun*. **5**, doi:10.1038/ncomms4646 (2014). 10.1038/ncomms4646 24736470

[8] Nozik AJ (2012). Photovoltaics: Separating multiple excitons. Nat. Photon..

[9] Ferrari AC, Basko DM (2013). Raman spectroscopy as a versatile tool for studying the properties of graphene. Nat. Nano..

[10] Thomsen C, Reich S (2000). Double resonant Raman scattering in graphite. Phys. Rev. Lett..

[11] Berkdemir A (2013). Identification of individual and few layers of WS_2_ using Raman spectroscopy. Sci. Rep..

[12] del Corro E (2014). Excited excitonic states in 1l, 2l, 3l, and bulk WS_2_ observed by resonant Raman spectroscopy. ACS Nano.

[13] Gaur A, Sahoo S, Scott J, Katiyar R (2015). Electron–phonon interaction and double-resonance Raman studies in monolayer WS_2_. J. Phys. Chem. C.

[14] Wang G (2015). Double resonant Raman scattering and valley coherence generation in monolayer WS_2_. Phys. Rev. Lett..

[15] del Corro E (2016). Atypical exciton-phonon interactions in WS_2_ and WSe_2_ monolayers revealed by resonance Raman spectroscopy. Nano Lett..

[16] Venezuela P, Lazzeri M, Mauri F (2011). Theory of double-resonant Raman spectra in graphene: Intensity and line shape of defect-induced and two-phonon bands. Phys. Rev. B.

[17] Herziger F (2014). Two-dimensional analysis of the double-resonant 2d Raman mode in bilayer graphene. Phys. Rev. Lett..

[18] Ferrari AC, Robertson J (2004). Raman spectroscopy in carbons: from nanotubes to diamond. Phil. Trans. R. Soc. A.

[19] Weber MC (2016). Temperature evolution of the band gap in BiFeO_3_ traced by resonant Raman scattering. Phys. Rev. B.

[20] Go S, Bilz H, Cardona M (1975). Bond charge, bond polarizability, and phonon spectra in semiconductors. Phys. Rev. Lett..

[21] Cardona M, Allen PB (1985). Theory of the resonant Raman scattering by two phonons in germanium and silicon. Helvetica Physica Acta.

[22] Trallero-Giner C, Cantarero A, Cardona M (1989). One-phonon resonant Raman scattering: Frölich exciton-phonon interaction. Phys. Rev. B.

[23] Grein CH, Zollner S, Cardona M (1991). Microscopic theory of second-order Raman scattering in silicon under uniaxial stress. Phys. Rev. B.

[24] Garcia-Cristobal A, Cantarero A, Trallero-Giner C, Cardona M (1994). Excitonic model for second-order resonant Raman scattering. Phys. Rev. B.

[25] Strauch D (1996). Full *ab initio* calculation of second-order infrared and Raman spectra of elemental semiconductors. Physica B.

[26] Renucci JB, Tyte RN, Cardona M (1975). Resonant Raman scattering in silicon. Phys. Rev. B.

[27] Knoll, P. & Ambrosch-Draxl, C. Raman scattering of atomic vibrations in anharmonic potentials. In *Proceedings of the International Workshop on Anharmonic Properties of High - Tc Cuprates*, 220 (World Scientific Publishing Co, Singapore, 1995). Eds. Mihailovic, G., Ruani, D., Kalids, E., and Müller, K. A.

[28] Ambrosch-Draxl C (2002). Raman scattering in YBa_2_Cu_3_O_7_: a comprehensive theoretical study in comparison with experiments. Phys. Rev. B.

[29] Puschnig P, Ambrosch-Draxl C, Henn RW, Simon A (2001). Electronic properties and Raman spectra of rare-earth carbide halides investigated from first principles. Phys. Rev. B.

[30] Ravindran P (2003). Raman- and infrared-active phonons in superconducting and non-superconducting rare-earth transition-metal borocarbides from full-potential calculations. Phys. Rev. B.

[31] Spitaler J, Sherman EY, Ambrosch-Draxl C (2007). Zone-center phonons in NaV_2_O_5_: A comprehensive *ab-initio* study including Raman spectra and electron-phonon interaction. Phys. Rev. B.

[32] Spitaler J, Sherman EY, Ambrosch-Draxl C (2008). First-principles study of phonons, optical properties, and Raman spectra in MgV_2_O_5_. Phys. Rev. B.

[33] Spitaler J, Sherman EY, Ambrosch-Draxl C, Evertz HG (2009). Lattice vibrations in CaV_2_O_5_ and their manifestations: A theoretical study based on density functional theory. New J. Phys..

[34] Gillet Y, Giantomassi M, Gonze X (2013). First-principles study of excitonic effects in raman intensities. Phys. Rev. B.

[35] Baroni S, Resta R (1986). *Ab initio* calculation of the low-frequency raman cross section in silicon. Phys. Rev. B.

[36] Baroni S, Giannozzi P, Testa A (1987). Green’s-function approach to linear response in solids. Phys. Rev. Lett..

[37] Gonze X (1995). Adiabatic density-functional perturbation theory. Phys. Rev. A.

[38] Baroni S, de Gironcoli S, Dal Corso A, Giannozzi P (2001). Phonons and related crystal properties from density-functional perturbation theory. Rev. Mod. Phys..

[39] Veithen M, Gonze X, Ghosez P (2004). First-principles study of the electro-optic effect in ferroelectric oxides. Phys. Rev. Lett..

[40] Veithen M, Gonze X, Ghosez P (2005). Nonlinear optical susceptibilities, raman efficiencies, and electro-optic tensors from first-principles density functional perturbation theory. Phys. Rev. B.

[41] Gonze X, Rignanese G-M, Caracas R (2005). First-principle studies of the lattice dynamics of crystals, and related properties. Z. Kristallogr..

[42] Caracas R, Bobocioiu E (2011). The wurm project—a freely available web-based repository of computed physical data for minerals. Am. Mineral..

[43] Gonze X, Lee C (1997). Dynamical matrices, born effective charges, dielectric permittivity tensors, and interatomic force constants from density-functional perturbation theory. Phys. Rev. B.

[44] Temple PA, Hathaway CE (1973). Multiphonon Raman spectrum of silicon. Phys. Rev. B.

[45] Wang CS, Chen JM, Becker R, Zdetsis A (1973). Second order Raman spectrum and phonon density of states of silicon. Phys. Lett..

[46] Trommer R, Cardona M (1977). Resonant Raman scattering by 2TO phonons and the ordering of conduction band minima in GaAs. Solid State Comm..

[47] Pasternak A, Cohen E, Gilat G (1974). Calculation of second-order Raman scattering for KBr, NaCl, and MgO crystals. Phys. Rev. B.

[48] Reich S (1995). Resonant Raman scattering in cubic and hexagonal boron nitride. Phys. Rev. B.

[49] Gatti M, Sottile F (2013). Exciton dispersion from first principles. Phys. Rev. B.

[50] Verdi C, Giustino F (2015). Fröhlich Electron-Phonon Vertex from First Principles. Phys. Rev. Lett..

[51] Hanke W, Sham LJ (1979). Many-particle effects in the optical excitations of a semiconductor. Phys. Rev. Lett..

[52] Onida G, Reining L, Rubio A (2002). Electronic excitations: density-functional versus many-body green’s-function approaches. Rev. Mod. Phys..

[53] Gonze X (2009). Abinit: First-principles approach of materials and nanosystem properties. Comput. Phys. Comm..

[54] Gonze X (2016). Recent developments in the ABINIT software package. Comput. Phys. Comm..

[55] Gonze, X. & Beuken, J.-M. ABINIT–abinit. http://www.abinit.org. (2016) (Date of access: 02/10/2016).

[56] Ambrosch-Draxl C, Sofo J (2006). 0. Linear optical properties of solids within the full-potential linearized augmented planewave method. Comp. Phys. Commun..

[57] Cappellini G, Del Sole R, Reining L, Bechstedt F (1993). Model dielectric function for semiconductors. Phys. Rev. B.

